# Novel Exopolysaccharide Produced from Fermented Bamboo Shoot-Isolated *Lactobacillus Fermentum*

**DOI:** 10.3390/polym12071531

**Published:** 2020-07-10

**Authors:** Thi Bich Thuy Do, Thi Ai Luyen Tran, Thi Van Thi Tran, Trung Hieu Le, Vijay Jayasena, Thi Hong Chuong Nguyen, Chinh Chien Nguyen, Soo Young Kim, Quyet Van Le

**Affiliations:** 1 Faculty of Engineering and Food Technology, Hue University of Agriculture and Forestry, Hue University, Thua Thien Hue 530000, Vietnam; 2 Hue Tourism College, Thua Thien Hue 530000, Vietnam; luyenqxqb@gmail.com; 3 University of Sciences, Hue University, Thua Thien Hue 530000, Vietnam; ttvthi@hueuni.edu.vn (T.V.T.T.); lthieu@hueuni.edu.vn (T.H.L.); 4 School of Science and Health, Western Sydney University, NSW 2751 Penrith, Australia; v.jayasena@westernsydney.edu.au; 5 Institute of Research and Development, Duy Tan University, Da Nang 550000, Vietnam; hongchuong1991@gmail.com; 6 Faculty of Environmental and Chemical Engineering, Duy Tan University, Da Nang 550000, Vietnam; 7 Department of Materials Science and Engineering, Korea University, 145 Anam-ro, Seongbuk-gu, Seoul 02841, Korea

**Keywords:** lactic acid bacteria, phenylalanyl-tRNA synthase gene, exopolysaccharide, gas–liquid chromatography–mass spectrometry, *Lactobacillus fermentum*, nuclear magnetic resonance spectroscopy

## Abstract

This study aimed at providing a route towards the production of a novel exopolysaccharide (EPS) from fermented bamboo shoot-isolated *Lactobacillus fermentum*. A lactic acid bacteria strain, with high EPS production ability, was isolated from fermented bamboo shoots. This strain, R-49757, was identified in the BCCM/LMG Bacteria Collection, Ghent University, Belgium by the phenylalanyl-tRNA synthetase gene sequencing method, and it was named *Lb. fermentum* MC3. The molecular mass of the EPS measured via gel permeation chromatography was found to be 9.85 × 10^4^ Da. Moreover, the monosaccharide composition in the EPS was analyzed by gas chromatography–mass spectrometry. Consequently, the EPS was discovered to be a heteropolysaccharide with the appearance of two main sugars—D-glucose and D-mannose—in the backbone. The results of one-dimensional (1D) and two-dimensional (2D) nuclear magnetic resonance spectroscopy analyses prove the repeating unit of this polysaccharide to be [→6)-β-D-Glc*p*-(1→3)-β-D-Man*p*-(1→6)-β-D-Glc*p*-(1→]*_n_*, which appears to be a new EPS. The obtained results open up an avenue for the production of novel EPSs for biomedical applications.

## 1. Introduction

Lactic acid bacteria (LAB) have attracted increasing attention in the food production of probiotics, owing to their benefits to human and animal health [1,2]. Thus, LAB are capable of not only reducing the risk of diarrhea, but also producing enzymes to support the digestive process, thereby reducing the risk of gastrointestinal disorders [3,4]. Furthermore, Saikali et al. [5] and Thirabunyanon et al. [6] discovered that fermented milk products containing probiotics reduce the risk of colon cancer, which highlights the compelling potential of LAB in cancer therapies. Additionally, many LAB strains have demonstrated exopolysaccharide (EPS) production capabilities. These EPSs exhibit good rheological properties, such as viscosity, emulsion stability, and gelling, and they act as prebiotics [1,2,7,8,9,10,11]. Extensive studies on LAB isolation and identification have been conducted in the past decades. The identification methods include traditional [12] as well as molecular biotechnological methods, such as 16S rRNA sequencing [13,14,15], phenylalanyl synthetase (*pheS*) and ribonucleic acid (RNA) polymerase enzyme (*rpoA*) sequencing [16,17], repetitive extragenic palindromic sequence-based polymerase chain reaction (rep-PCR) fingerprinting [14,18], (GTG)_5_-PCR [19], and matrix-assisted laser desorption/ionization-time of flight (MALDI-TOF) mass spectrometry (MS) [14,20,21]. Traditional methods are time-consuming, because they are based on numerous biochemical reactions of strains, their optimum growth conditions (e.g., pH, NaCl concentration, and temperature), cell, and colony morphology. In addition, the results of these methods are occasionally incompletely exact.

Numerous efforts have been devoted towards the enhancement of the EPS yield. The amount of EPS produced in a medium depends on various factors, such as temperature, pH, medium composition, and incubation time. Furthermore, EPS quality is influenced by the LAB species. The amounts of EPS produced by *Lb. fermentum* TDS030603 [22], *Lb. fermentum* F6 [23], and *Leu. Pseudomesenteroides* [24] were 97.1 mg/L, 33.05 mg/L, and 12.5 g/L, respectively. The molecular weights of the EPS were 1 × 10^5^ Da for *Lb. johnsonii* 142 [25], 1.7 × 10^5^ Da for *Lb. plantarum* 70810 [9], 1.03 × 10^5^ Da for *Lb. plantarum* YW32 [8], 1.15 × 10^6^ Da for *Lb. plantarum* C88 [26], and 5 × 10^6^ Da for *Lb. delbrueckii* subsp. *bulgaricus* OLL1073R-1 [27]. LAB diversity and the different EPS characteristics have attracted many scientists to focus on the EPS production of these bacteria.

EPS structures that are produced by LAB fermentation are diverse, and they depend on LAB species. This diversity includes the different sugar monomers and glycosidic linkages present in the repeating units. The EPS produced by *Lb. delbrueckii* ssp. *bulgaricus* LBB.B26 contains mixed monosaccharides of glucose and galactose with a major branched pentasaccharide repeating unit of →3)-α-D-Gal*p*[α-D-Glc*p*(1→6)]-α-D-Gal*p*(1→4)- α -D-Glc*p*(1→3)- α -D-Gal*f*(1→ [28]. The main EPS backbone from *Lb. fermentum* TDS030603 was reported by [29], comprising 1→3)- α -D-glucans and low content branches of α-D-Glc*p*(1→ and →6)α-D-Gal*p*(1→. Yang et al. [24] showed that the EPS produced by *Leuconostoc pseudomesenteroides* YF32 was a glucan with a peak—a linear backbone composed of consecutive α-(1→6)-linked D-glucopyranose units. Glucan-secreted *Leuconostoc lactis* KC117496 contains 95% of α-(1→6) [30]. Thus far, the production of EPS from *Lactobacillus* fermentum isolated from fermented bamboo shoots is practically non-existent and, therefore, an urgent investigation is essential.

Inspired by these challenges, herein we offer a novel route to the production of LAB species isolated from fermented bamboo shoots and the determination of EPS production abilities and characterizations. Impressively, by employing advanced techniques to identify the EPS structure (e.g., gas chromatography-mass spectrometry (GC-MS), as well as one-dimensional (1D) and two-dimensional (2D) nuclear magnetic resonance (NMR), a novel EPS—[→6)-β-D-Glc*p*-(1→3)-β-D-Man*p*-(1→6)-β-D-Glc*p*-(1→]*_n_*—has been discovered for the first time.

## 2. Materials and Methods

### 2.1. Material

Ten samples each of fermented bamboo shoots and fermented meat, named “nem chua”, were aseptically collected from households throughout Hue city in Vietnam. The samples were kept at 4 °C for analysis.

### 2.2. Isolation of Lactic Acid Bacteria

To isolate the LAB, each sample (15 g) was ground aseptically, suspended in Ringer’s solution (135 mL) (Sigma–Aldrich, Milan, Italy), and homogenized in a stomacher (BagMixer 400; Interscience, Saint Nom, France) for 2 min. at maximum speed. The homogenized solution was then serially diluted. Decimal dilutions were plated on de Man, Rogosa, and Sharpe (MRS) agar (Oxoid, Milan, Italy) and incubated under anaerobic conditions at 37 °C for 48 h. After incubation, the colonies (10–15 colonies for each sample) were randomly selected and plated on the MRS agar until pure cultures, identified by colony morphology, were formed. All of the isolates then underwent Gram staining and catalase reactions. Only Gram-positive and catalase-negative samples were selected as LAB and stored in Microbank^TM^ vials (Pro-Lab Diagnostics, Richmond Hill, ON, Canada) at −80 °C for further analysis.

### 2.3. Screening Isolate Having High Ability of EPS Production

The EPS biosynthesis ability of LAB was determined by growing the strains that were isolated from the fermented bamboo shoots (MC2, MC3) and “nem chua” (N9, N10) on MRS. After incubation at 37 °C, with an initial pH value of 6.0–6.2 for 48 h, the cultures were boiled at 100 °C for 10 min. After cooling, they were treated with trichloroacetic acid, and the cells and protein were removed by centrifugation (10,000× *g* for 10 min. at 4 °C, Centrifuge model K2015R) twice. Next, the EPS in the supernatant was retained by means of cold ethanol precipitation at 4 °C for 24 h. The amount of EPS was determined via the phenol-sulfuric method [31] while using glucose as the standard. Briefly, a mixture of reactions containing 1 mL of EPS solution, 1 mL of phenol 5%, and 5 mL of concentrated sulfuric acid was vortexed and streamed for 2 min. Thereafter, the mixture was placed at room temperature for 30 min. The absorbance of the characteristic yellow-orange color was measured at 490 nm, and the blank was prepared by substituting distilled water for the EPS solution.

### 2.4. EPS Production and Purification

*Lb. fermentum* MC3 was cultured in MRS with 4.0% glucose and 0.3% yeast extract. After incubation at 37 °C with an initial pH of 6.0–6.2 for 48 h, the cultures were boiled at 100 °C for 10 min. After cooling, they were treated with trichloroacetic acid, and the cells and protein were removed by centrifugation (10,000× *g* for 10 min. at 4 °C, Centrifuge model K2015R) twice. Thereafter, the EPS in the supernatant was retained via cold ethanol precipitation at 4 °C for 24 h.

For purification, 10 mL of EPS crude solution with a concentration of 10 mg/mL was added into a 26 nm × 500 mm diethylaminoethyl (DEAE)-cellulose-52 anion-exchange chromatography column. Sample elution was performed at a flow rate of 1 mL/min. with deionized water, as well as 0.1, 0.3, and 0.5 M of NaCl solution. The EPS fraction that was collected after exchange chromatography (10 mL) was then purified through a 10 mm × 600 mm Sephadex G-100 column. The flow rate in this process was 0.2 mL/min. with deionized water. The purified EPS samples for further analyses were obtained after dialyzing and lyophilizing the EPS fractions from the Sephadex G-100 column [32].

### 2.5. Identification of LAB by Phenylalanyl-tRNA Synthase (pheS) Gene Sequencing

#### 2.5.1. Preparing DNA Extracts

The genomic deoxyribonucleic acid (DNA) extraction of the bacterial isolates was performed using an alkaline lysis buffer as described by Birnboim [33]. One colony of each sample was incubated in a 1.5 mL tube with a 20 µL alkaline lysis buffer containing 0.05 mol·L^−1^ NaOH and 0.25% safety data sheet at 95 °C for 15 min. and then placed on ice. After brief spinning, 180 µL of Milli-Q water was added into the tube, centrifuged for 3 min. at 13,000 rpm, and then stored at −20 °C for further analysis.

#### 2.5.2. PCR and Sequencing

The set of primers *phe*S-21-F (5′-CAYCCNGCHCGYGAYATGC-3′) (forward primer) and *phe*S-23-R (5′-GGRTGRACCATVCCNGCHCC-3′) (reverse primer) was used for polymerase chain reaction (PCR) amplification. The PCR for each sample was composed of 16.5 µL of sterile Milli-Q water, 2.5 µL of PCR buffer 10×, 2.5 µL of deoxynucleoside triphosphates (dNTPs), 0.25 µL of forward primer (50 µM), 0.25 µL reverse primer (50 µM), 0.5 µL of AmpliTaq DNA polymerase (1 U/µL), and 2.5 µL of alkaline lysis DNA. PCR was performed using the Veriti thermal cycler (Life technologies). The thermal program consisted of (1) 5 min. at 95 °C, (2) 3 cycles of 1 min. at 95 °C + 2 min. 15 s at 50 °C + 1 min. 15 s at 72 °C, (3) 30 cycles of 35 s at 95 °C + 1 min. 15 s at 50 °C + 1 min. 15 s at 72 °C, and (4) a final 7 min. at 72 °C. The PCR products were analyzed by RESult 1% LE Agarose (Biozym, The Netherlands) gel electrophoresis [16] to confirm the LAB strain.

The Nucleofast 96 PCR clean-up membrane system (Machery–Nagel, Germany) was applied to purify the products of positive PCRs. These PCR samples were loaded into the wells with ultrafiltration membranes of a filter plate. Under a vacuum pressure of up to −0.6 bar, the contaminants (primers, dNTPs, and salts) were filtered to waste. The desired PCR products that were retained on the membrane were washed by adding 100 µL of sterile Milli-Q water and then filtered. For recovery, the PCR products were then eluted in 70 µL of sterile Milli-Q water. These products were used for sequencing. Subsequently, 3.0 µL of the purified and eluted PCR product was mixed with 4 µL of ABI Prism BigDye Terminator Cycle Sequencing Ready Reaction Mix version 3.1 (Applied Biosystems, Foster City, CA, USA), 3.0 µL of sequencing primer (4 µM), 1.5 µL 56 dilution buffer, and 1.5 µL of Milli-Q water. The primers used for this sequencing were *phe*S-21-F and *phe*S-23-R. The thermal program consisted of 30 cycles of 15 s at 96 °C + 1 s at 35 °C + 4 min. at 60 °C. Sequencing products were purified using a BigDye XTerminator Purification kit (Applied Biosystems, Life Technologies) according to the manufacturer’s instructions. The DNA fragments were separated in an ABI PRISM 3130XL genetic analyzer (Applied Biosystems, Foster City, CA, USA). The time and voltage of sample injection were 20 s at 1.25 kV, and each run was performed at 50 °C for 6500 s at 0.1 mA and 12.2 kV.

#### 2.5.3. Sequence Analysis

The produced electropherograms were analyzed via sequencing analysis in the BioNumerics 7 software (Applied Maths). Sequences were determined using two reads of *pheS* gene. The National Center for Biotechnology Information (NCBI) Basic Local Alignment Search Tool (BLAST) (www.ncbi.nlm.nih.gov/BLAST) was used for analysis of the *pheS* gene sequences.

### 2.6. Estimation of EPS Molecular Mass

The average molecular weight of the EPS produced by *Lactobacillus fermentum* MC3 (EPS-MC3) was determined by gel permeation chromatography (GPC—Agilent 1100, USA), as described by Fukuda et al. (2010) with some modifications. The purified EPS was dissolved in 0.1 M (10 µL) NaNO_3_ and injected in the system (Agilent 1100 Series coupled to MS detector, microTOF-QII Bruker) by maintaining the same flow rate and column temperature. Separation was carried out using 0.1 M NaNO3 as the mobile phase, and pullulan was used as the standard with known molecular masses, including 5, 10, 20, 50, 100, 200, 400, and 800 kDa. These standards were loaded onto a Ultrahydrogen 500 column (7.8 mm × 300 mm, 10 µm). Elution was done with 0.1 M NaNO3 at 40 °C and a flow rate of 1 mL/min.

### 2.7. Monosaccharide Composition and Methylation Analysis

#### 2.7.1. Methylation Analysis

The polysaccharide samples were methylated using methyl disulfate and solid sodium hydroxide for 16 h in dimethyl sulfoxide (DMSO) at 60 °C.

#### 2.7.2. EPS Hydrolysis

Five milligrams of methylated EPS were hydrolyzed for monosaccharide composition analysis with 4 mL of trifluoroacetic acid (TFA) 2 M (2 h, 120 °C), followed by evaporation under a stream of N_2_. Excess TFA was removed by co-evaporation with MeOH under a stream of N_2_.

#### 2.7.3. Converting

Converting monosaccharides into alditol acetates: the resulting partially methylated monosaccharides were reduced with 0.25 M NaBH4 in NH_3_ (30 min., room temperature). The solution was neutralized with 5 mL of acetic acid (10%) in MeOH, lyophilized, and the boric acid was removed by co-evaporation with MeOH under a stream of N_2_.

#### 2.7.4. Acetylation

Acetylation for GC-MS: the samples were acetylated with 2 mL of anhydride acetic: pyridine (1:1, v/v) at 100 °C for 20 min. The mixtures of partially methylated alditol acetates were dried under a stream of nitrogen. The resulting products were dissolved in ethyl acetate and were analyzed by GC–MS [34].

#### 2.7.5. GC-MS

GC-MS system (Shimadzu 2010): the temperature was programmed to be 150 °C for 1 min., followed by 250 °C for 10 min., and then 280 °C for 5 min. The total time was approximately 20 min.

### 2.8. NMR Method

A solution of the polysaccharide (50 mg) in 2 M TFA was kept for 5 h at 75 °C, or partial acid hydrolysis, and then lyophilized. Samples (10 mg) were dissolved in DMSO (1 mL). Spectra were recorded at 302.5 K and 302.9 K on a Bruker Avance 500 Hz spectrometer using trimethylsilane as an internal reference. NMR spectrum was recorded at 500 MHz for ^1^H and at 125 MHz for ^13^C NMR. The 2D spectra (heteronuclear single-quantum correlation spectroscopy (HSQC), correlation spectroscopy (COSY), heteronuclear multiple-bond correlation spectroscopy (HMBC), and nuclear Overhauser effect spectroscopy (NOESY)) were reported to determine the sugar residues. Chemical shifts (δ) were given in parts per million (ppm).

### 2.9. Statistical Analysis

The data were statistically analyzed using the one-way ANOVA procedure of SPSS (version 20.0) and expressed as mean ± SD. All of these experiments were performed in triplicate and within each replication; analyses were carried out in duplicate. The differences among means were tested by the Student Newman–Keuls test. Data were considered statistically significant when *p* < 0.05.

## 3. Results and Discussion

### 3.1. Ability of EPS Production of Isolates

**Figure 1:**
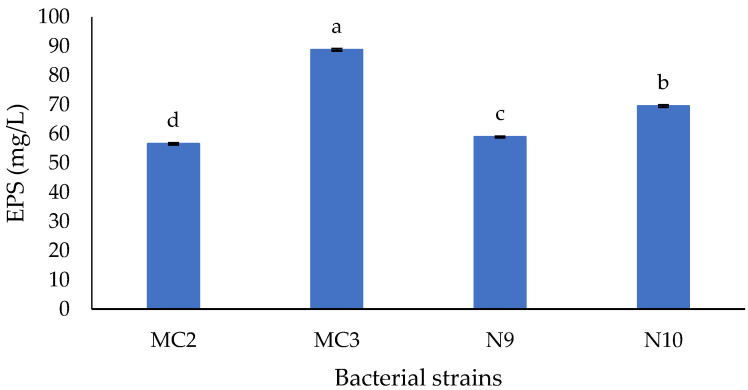
The production of exopolysaccharide (EPS) from some bacterial strains of fermented bamboo shoots and fermented meat. Data are means SD of EPS production from triplicate experiments. Bars with different letters are significantly different at *p* < 0.05.

MC3 produced the highest EPS amount in MRS broth at 37 °C for a 48-h incubation. The EPS yield from MC3 was 88.776 mg/L (Figure 1). The growth of MC2, N9, and N10 strains under the cultivation conditions used generated poor EPS yields of 56.581, 58.939, and 69.508 mg/L, respectively.

The results showed that the amount of EPS produced by the MC3 strain is significantly higher than that produced by the other strains (Figure 1). This can be attributed to various factors, such as the age, physiological characteristics of each strain, and enzyme activity of EPS biosynthesis. The MC3 strain was selected for further characterization by the *PheS* gene sequencing method.

From the result of *Phe*S sequencing, the MC3 strain was identified as *Lactobacillus fermentum* (*Lb. fermentum*) with a similarity of 100% with the NCBI accession numbers of referent species, sCP025592.1 and CP017712.1. Its strain number is R-49757 in the BCCM/LMG Bacteria collection, Ghent University, Belgium.

### 3.2. Average Molecular Weight

**Figure 2:**
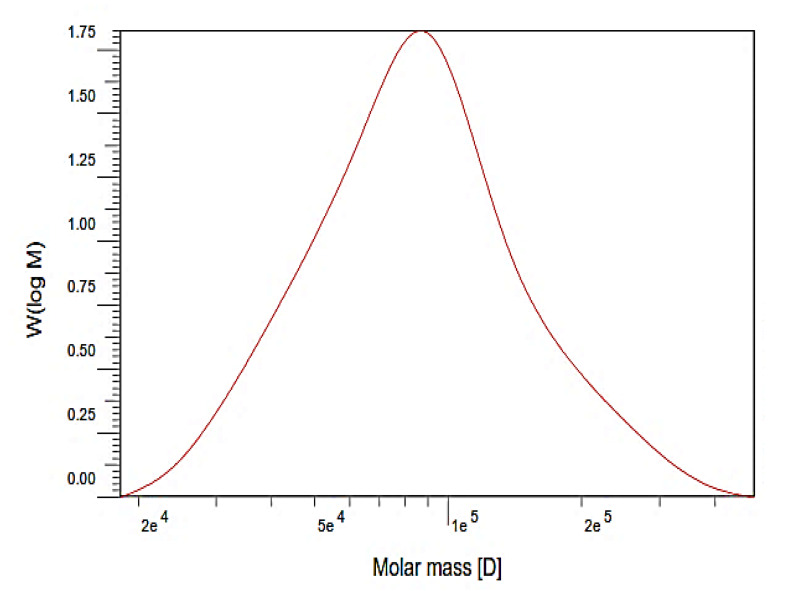
The molecular mass chromatogram of EPS from Lb. fermentum MC3 obtained by gel permeation HPLC MC3.

The chromatogram of EPS-MC3 obtained by gel-permeation high performance liquid chromatography (HPLC) depicted a single peak of weight average molecular weight (MW) (Figure 2). The average molecular weight of EPS-MC3 in modified MRS was approximately 9.85 × 10^4^ Da. The EPS-MC3 is heterogeneous EPS with the polydispersity index value of 1.35, which is determined from the ratio of MW to number average molecular weight (*M*n).

The average molecular weight of the EPS produced by the *Lb. fermentum* TDS030603 strain was lower than that produced by *Lactobacillus fermentum* MC3. Fukuda et al. (2010) reported that the molecular mass of EPSs produced by *Lb. fermentum* TDS030603 has similar values when grown in MRS and in other media with different carbohydrate sources [22]. The EPSs contained lower molecular mass fractions (approximately 45 kDa) and higher molecular weight fractions (200 and 550 kDa) [35]. By means of the gel-permeation chromatography technique, the values of average molecular weights of EPSs, including EPS from *Lactobacillus helveticus* (LB1 and LB2) and c-EPS from *Lb. plantarum* 70810, were estimated to be 5.4 × 10^5^ Da and 20.3 × 10^5^ Da, respectively [36]; and, 169.6 kDa [32].

### 3.3. Methylation Analysis of EPS-MC3

GC-MS analysis of the monosaccharide composition of EPS-MC3 showed that the EPS was a heteropolysaccharide with the appearance of two main methylated sugar derivatives—1,5,6-tri-O-acetyl-2,3,4-tri-O-methyl-glucitol and 1,3,5-tri-O-acetyl-2,4,6-tri-O-methyl-mannitol—supposedly owing to the presence of D-glucose and D-mannose in the backbone. It was shown that there were two types of linkages in the EPS from *Lb. fermentum* MC3: (1 → 6)-linked glucosyl and (1 → 3)-linked mannosyl.

Previous studies have reported on the presence of glucose in the monosaccharide composition of EPSs from *fermentum* species. Glucose and galactose in EPS produced by *Lb. fermentum* TDS030603 [29]; glucose, rhamnose, and galactose in EPS produced by *Lb. fermentum* V10 [37]; and, the EPS secreted by *Lb. fermentum* Lf2 contained glucose and galactose [38]. There are no published data showing the presence of mannose in the repeating unit of EPSs from *Lb. fermentum* species. It can be concluded that the EPS from *Lb. fermentum* MC3 is novel. The differences between the monosaccharide compositions of EPSs could be related to the age differences, enzyme activity in the EPS biosynthesis, and the cultivation conditions.

### 3.4. ^1^H NMR and ^13^C NMR Analysis of EPS-MC3

**Figure 3:**
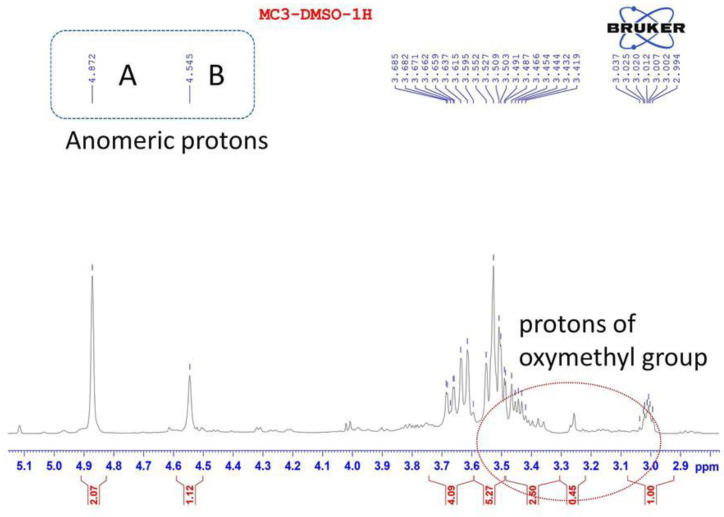
^1^H NMR spectrum of EPS from Lb. fermentum MC3.

The ^1^H NMR spectrum of EPS produced by *Lb. fermentum* MC3 contains two resonances in the region of anomeric proton at δ 4.87; 4.55 ppm, and anomeric region (δ 3.00–3.62), which are protons of oxymethyl groups. The anomeric signals of the ^1^H-NMR spectrum revealed the presence of disaccharide repeating units in the EPS-MC3 structure. These residues are designated as A and B according to decreasing chemical shift values of the anomeric protons (Figure 3). The chemical shifts of anomeric protons (less than 5.0 ppm) are typical of those of the anomeric protons of β-linked residues [22,27,29,39]. The values of the H_1_ proton exceeded 5 ppm, indicating that these were α-type configurations. Figure 3 shows that the signals of anomeric protons of EPS-MC3 are δ 4.87 ppm and δ 4.55 ppm. Therefore, EPS from *Lb. fermentum* MC3 only contained β-type glycosidic linkages.

**Figure 4:**
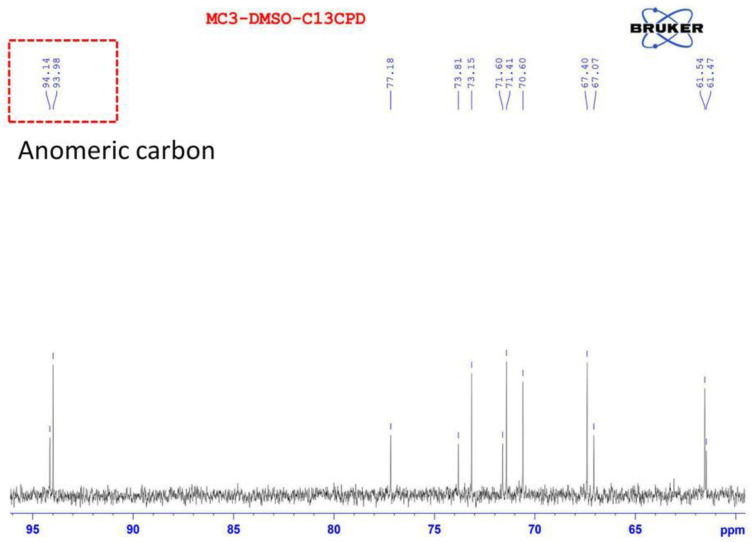
^13^C NMR spectrum of EPS from *Lb. fermentum* MC3.

The result of the ^13^C NMR spectrum (Figure 4) shows that there are two signals of anomeric carbon with chemical shift values at 94.1 and 94.0 ppm. The major chemical shift signals for 93–94.5 ppm were found in ^13^C NMR, and they are probably for glucose and mannose [39,40].

The anomeric resonance signal at 94.0 ppm could be for β-D-glucopyranose and the other chemical shift of anomeric carbon (94.1 ppm) is for β-D-mannopyranose (Figure 4). The NMR data indicated the presence of glucose and mannose in the repeating unit of EPS-MC3. According to GC-MS, glucose and mannose were discovered to be monosaccharides. 2D NMR spectrums. further characterized the detailed chemical structure of EPS produced by the *Lb. fermentum* MC3 strain.

**Figure 5:**
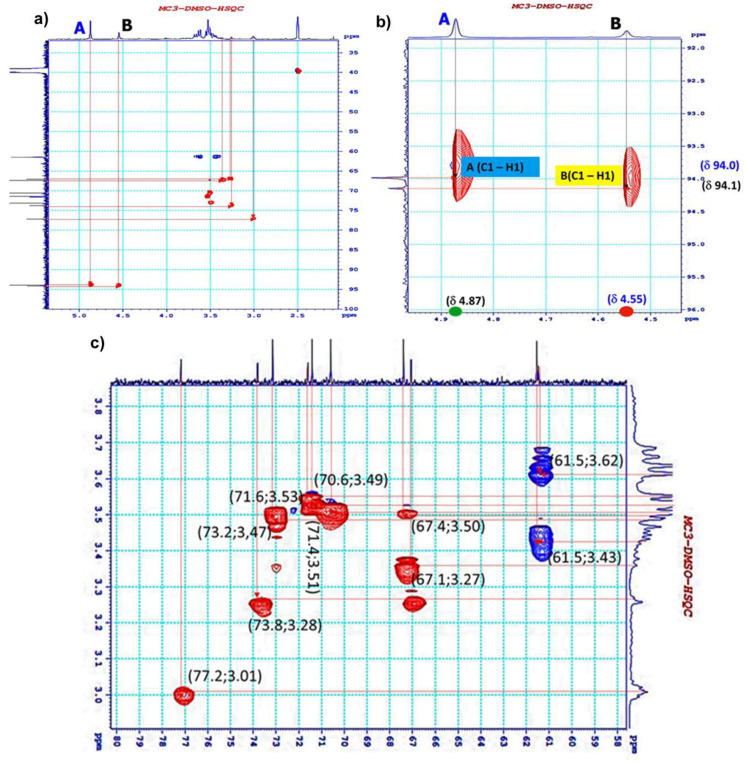
2D ^1^H–^13^C heteronuclear single-quantum correlation spectroscopy (HSQC) spectrum of EPS from Lb. fermentum MC3; a (overall); b (expansion 1); and, c (expansion 2).

The ^1^H–^13^C HSQC spectrum showed that there were 12 cross-peaks confirmed in the region for anomeric resonances δ_C_ 61.5–94.1 ppm and δ_H_ 3.62–4.55 ppm (Figure 5). Residue A was indicated by the signals at δ 4.87/90.4, δ 3.53/71.6, δ 3.62/61.5, δ 3.49/70.6, δ 3.28/73.8, and δ 3.51/71.4. The presence of the signals at δ 4.55/94.1, δ 3.01/77.2, δ 3.47/73.2, δ 3.27/67.1, δ 3.50/67.4, and δ 3.43/61.5 indicated by residue B. The number of cross-peaks in the region for methylene groups implied that the monosaccharides were in hexose forms.

**Figure 6:**
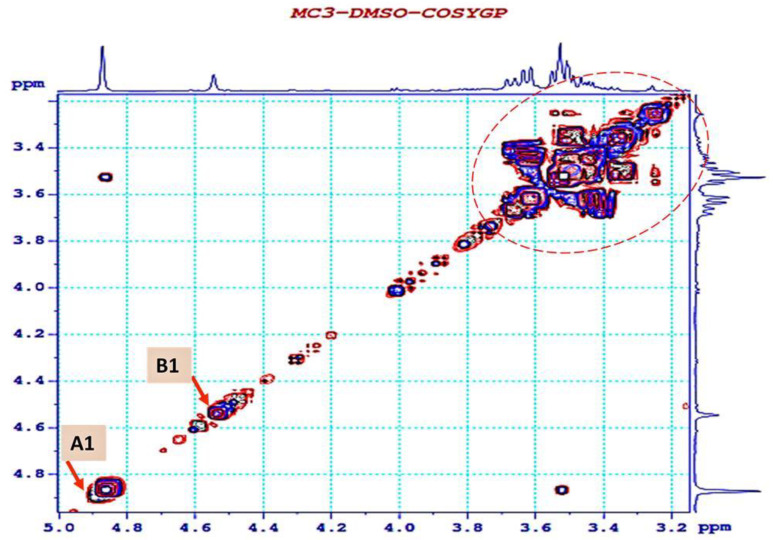
2D ^1^H–^1^H correlation spectroscopy (COSY) spectrum of EPS from Lb. fermentum MC3.

The coupling of the anomeric proton ^1^H–^1^H COSY spectra of A and B between A H-2 (δ 3.53)/A H-3 (δ 3.62)/A H-4 (δ 3.49), A H-4 (δ 3.49)/A H-3 (δ 3.62)/ A H-5 (δ 3.28) and between B H-3 (δ 3.47)/B H-4 (δ 3.27)/B H-5 (δ 3.50), B H-5 (δ 3.50)/B H-4 (δ 3.27)/B H-6 (δ 3.43) were observed (Figure 6). These data permitted the setting of the carbon linkage sequence of the monosaccharides in the EPS-MC3 structure.

The combination of results of ^1^H NMR and ^13^C NMR, as well as the 2D COSY, HSQC, and NMR spectra, in addition to the reports of [39,41,42,43,44], can be used to determine the chemical shift of the sugar residues, as shown in Table 1.

**Figure 7:**
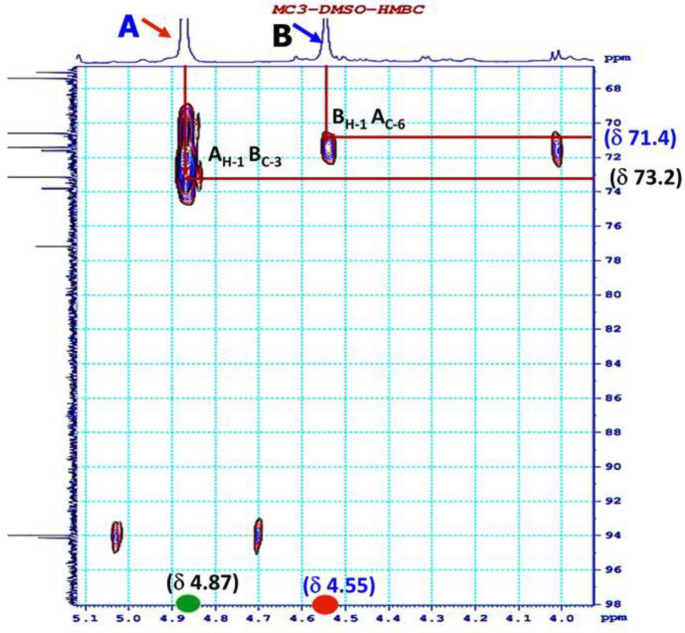
Heteronuclear multiple-bond correlation spectroscopy (HMBC) spectrum of EPS from Lb. fermentum MC3.

The HMBC spectra showed an inter-residue cross-linking between the anomeric proton and the carbon at the linkages between A H-1 and B C-3; B H-1 and A C-6 (Figure 7). These linkages confirmed the presence of A(1→3)B and B(1→6)A bonding.

**Figure 8:**
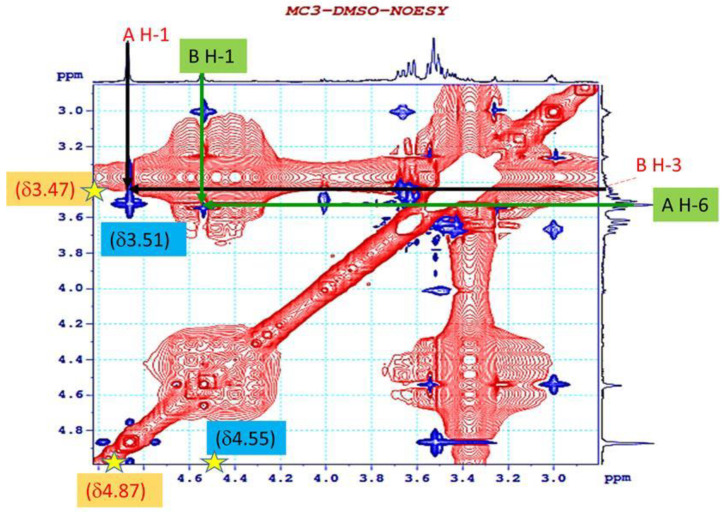
Nuclear Overhauser effect spectroscopy (NOESY) spectrum of EPS from Lb. fermentum MC3.

In addition, the sequence and linkages between the sugars were confirmed with the NOESY spectra, as shown in Figure 8. The strong cross peaks were found between H-1 (δ 4.87) of A and H-3 (δ 3.47) of B, leading to the assignment of a A(1→3)B linkage; H-1 (δ 4.55) of B and H-6 (δ 3.51) of A, which supported the occurrence of a B(1→6)A linkage in the repeating unit.

The HMBC and NOESY data identified the presence of two linkages, which are A(1→3)B and B(1→6)A, in the repeating unit of EPS-MC3. The data are summarized in Table 2.

The results of the 1D and 2D NMR analyses indicate that the EPS from *Lb. fermentum* MC3 strain has the following repeating unit structure: [→6)-β-D-Glc*p*-(1→3)-β-D-Man*p*-(1→6)-β-D-Glc*p*-(1→]*_n_*.

*Lb. fermentum* was found to constitute 19% of isolates from tarhana, a traditional fermented product in Turkey. These strains were identified by a combination of methods, including rep-PCR fingerprinting, multiplex PCR, 16S rRNA gene sequencing, and carbohydrate assimilation profiling [45]. The methods of phenotypic parameters, biochemical tests, and 16S rDNA gene sequencing were combined in order to identify a group of LAB isolated from Kahudi, a fermented mustard product of Assam, India. The result revealed that *Lb. fermentum* was one of the dominant LAB groups in this product [46]. *Lb. fermentum* was reported to occupy 7% in 273 LAB isolates from “nem chua,” a fermented meat product in Vietnam, when identified by combining (GTG)5-PCR fingerprinting, pheS, and *rpoA* gene sequence analysis [47].

The EPS biosynthesis capabilities of LAB depend on the strain. A lower yield (280 mg/L) of EPS was produced by *Lb. helveticus* ATCC 15807 when cultured in MRS at 30 °C, pH 4.5 [48]. Conversely, the *Lb. fermentum* CFR 2195 strain produced a higher EPS amount (28820 mg/L) from MRS with the supplement of sucrose (50.1 g/L). The EPS yield from MC3 was 88.776 mg/L, whereas its yield that was obtained from CFR was 28.85 g/L with a consumption of 18.7 g/L of sucrose in the medium after 24 h of incubation [49]. The reason could be related to various factors such as age, physiological characteristics of strains, and enzyme activity in the EPS biosynthesis. In this study, the MC3 strain was named as *Lb. fermentum* MC3 by the *pheS* gene sequencing method and further identified.

The molecular weights of the EPSs produced by LAB have a wide range of 10^5^–10^6^ Da for homopolysaccharides and 10^4^–6 × 10^6^ for heteropolysaccharides. The result obtained by Zhou et al. (2016) implied that the presence of monosaccharides in repeating units of EPS-A was higher than that in EPS-B, leading to a lower molecular weight of EPS-B as compared to EPS-A. The molecular weights of EPS-A and EPS-B were 3.97 × 10^5^ Da and 3.86 × 10^5^ Da, respectively [44]. The molecular weight of EPS can be high or low because of the monosaccharide compositions. EPS from *Lb. fermentum* MC3 produced a polysaccharide with a molecular weight of 9.85 × 10^4^ Da. Analysis of the sugar composition indicated that the EPS from *Lb. fermentum* MC3 was composed of glucose and mannose in a molar ratio of 1.00:0.91, which could cause their repeating unit to exhibit high levels in EPS biosynthesis.

**Figure 9:**
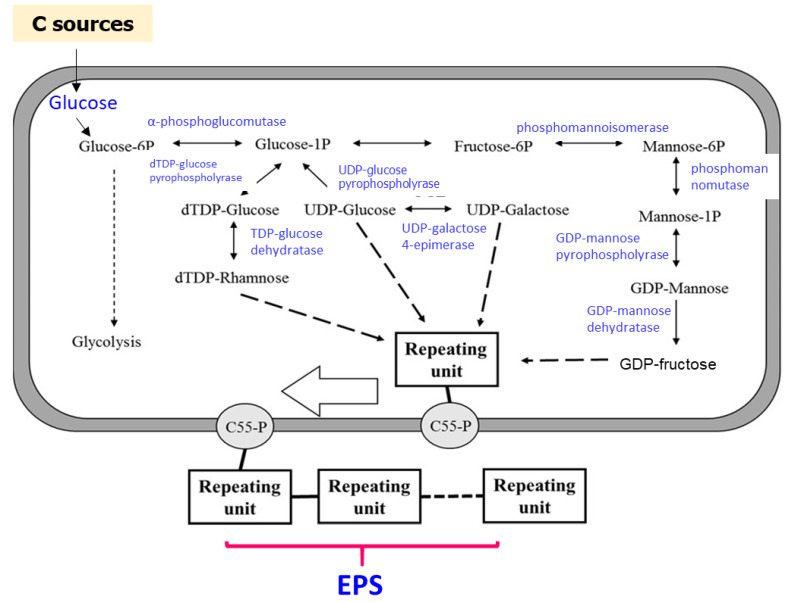
Outline of the biosynthesis of hetero EPS by lactic acid bacteria (LAB).

The presence of mannose in the EPS structure produced by *Lb. fermentum* MC3 is a new finding. The monosaccharide compositions of EPS from *Lb. fermentum* strains found in the previous studies were mainly glucose and galactose [29,38] or glucose, rhamnose, and galactose [37]. The appearances of different monosaccharides in the repeating unit structure of EPS are attributable to the enzyme activities in EPS production. Glucose is always found in EPS from *Lactobacillus* genus (Figure 9) [1,50]. This could be owing to the presence of uridine diphosphate-glucose pyrophosphorylase and uridine diphosphate-glucose dehydrogenase, which are enzymes that participate in glucose nucleotide in EPS establishment. However, the presence of enzymes, such as phosphomannomutase, mannose-1-phosphate guanylyltransferase, and guanosine diphosphate-mannose pyrophosphorylase, as well as the absence of guanosine diphosphate-mannose dehydratase from fructose nucleotide, lead to the appearance of mannose residue in the EPS produced by *Lb. fermentum* MC3. In addition, the presence of mannose in repeating units can be related to enzyme activities. In this study, the activities of enzymes that facilitate the synthesis of fructose nucleotide are significantly lower than those that facilitate the synthesis of mannose nucleotide; thus, the resulting fructose residue does not appear in the EPS structure that is produced from *Lb. fermentum* MC3.

The detailed data from 1D and 2D EPS-MC3 NMR analyses of partial acid hydrolysates indicated that EPS-MC3 was a lined polysaccharide consisting of (1→6)-linked Glc and (1→3)-linked Man. This is a new EPS as its structure is different from those of EPSs of other *Lb. fermentum* found in the literature.

## 4. Conclusions

In summary, a novel EPS was generated and identified from fermented bamboo shoot-isolated *Lactobacillus fermentum*. The characterization methods disclose that EPSs from *Lb. fermentum* MC3 are composed of the same repeating units of glucose and mannose with a molecular mass in the range of 10^4^–6 × 10^6^ Da. Importantly, the 1D and 2D NMR results indicate a new EPS from *Lb. fermentum* MC3 consisting of the units [→6)-β-D-Glc*p*-(1→3)-β-D-Man*p*-(1→6)-β-D-Glc*p*-(1→]*_n_*. Such a novel structure is rarely reported elsewhere. This study offers a very potential pathway for the production of novel and highly efficient EPS for biomedical applications.

## Figures and Tables

**Table 1:** ^1^H and ^13^C NMR chemical shifts (δ, ppm) of EPS from *Lb. fermentum* MC3 recorded in DMSO at 80 °C.

	Sugar Residue	H-1	H-2	H-3	H-4	H-5	H-6
A	→6)-β- D-glucopyranoside-(1→	4.87	3.53	3.62	3.49	3.28	3.51
B	→3)-β-D- mannopyranoside-(1→	4.55	3.01	3.47	3.27	3.50	3.43
	**Sugar Residue**	**C-1**	**C-2**	**C-3**	**C-4**	**C-5**	**C-6**
A	→6)-β- D-glucopyranoside-(1→	94.0	71.6	61.5	70.6	73.8	71.4
B	→3)-β-D- mannopyranoside-(1→	94.1	77.2	73.2	67.1	67.4	61.5

**Table 2:** Inter-glycosidic correlations from the anomeric atoms observed in ^1^H, ^1^H NOESY, and ^1^H, ^13^C HMBC spectra of EPS from *Lb. fermentum* MC3.

Sugar Residue		δ_H1 (ppm)_	NOESY to	HMBC to	Connectivity
(1→6)-β-D-glucopyranoside	A	4.87			A: H_1_ to B: H_3_
			3.47	73.2	A: H_1_ to B: C_3_
(1→3)-β-D- mannopyranoside	B	4.55			B: H_1_ to A: H_6_
			3.51	71.4	B: H_1_ to A: C_6_
